# Muscle Abscess due to Salmonella Enterica

**DOI:** 10.5812/ircmj.6852

**Published:** 2013-07-05

**Authors:** Yasemin Akkoyunlu, Bahadir Ceylan, Meryem Iraz, Nuh Mehmet Elmadag, Turan Aslan

**Affiliations:** 1 Bezmialem Vakif University, Department of Infectious Diseases and Clinical Microbiology, Istanbul, Turkey; 2 Bezmialem Vakif University, Department of Orthopaedics and Traumatology, Istanbul, Turkey

**Keywords:** Salmonellosis Infections, Abscess, Arthritis, Rheumatoid

## Abstract

Non typhoidal Salmonellae spp. causes clinical symptoms especially in neonates, infants, aged and immunocompromised patients. Hematogenous dissemination may occur in complicated cases whereas the formation of abscess is rare. A 61-year old woman presented to our hospital with pain and a mass in her left arm, without fever and leukocytosis. She was using methotrexate, corticosteroids and quinine for rheumatoid arthritis. She had a history of cervix cancer and was given radiotherapy and chemotherapy 3 years ago. Upon physical examination and magnetic resonance imaging, the mass was considered as an abscess and was surgically drained. Salmonella enterica spp. enterica was yielded in the culture of the drainage material. Ceftriaxon 2g/day was started intramuscularly and continued for 4 weeks. Salmonellosis is usually a self-limited disease, generally restricted to gastrointestinal tract and acquired following food poisoning. Management of Salmonella abscess requires a combination of antibiotherapy, surgical drainage and eradication of primary foci.

## 1. Introduction

Non-typhoidal Salmonellae spp. generally causes a self-limiting enterocolitis in immunocompetent individuals. Up to 5% of these patients develop secondary bacteremia. This microorganism can persist in the gastrointestinal tract after diarrheal illness 1. So-called primary non-typhoidal Salmonellae bacteremia, without associated diarrhea, occurs mainly in at risk groups such as neonates, infants, aged and immunocompromised patients due to malignancy, steroid use, HIV infection, chronic renal and liver diseases, diabetes or inherited immunodeficiencies. Hematogenous dissemination may occur in complicated cases whereas the formation of abscess is rare (1, 2).

## 2. Case Presentation

**Figure 1.**
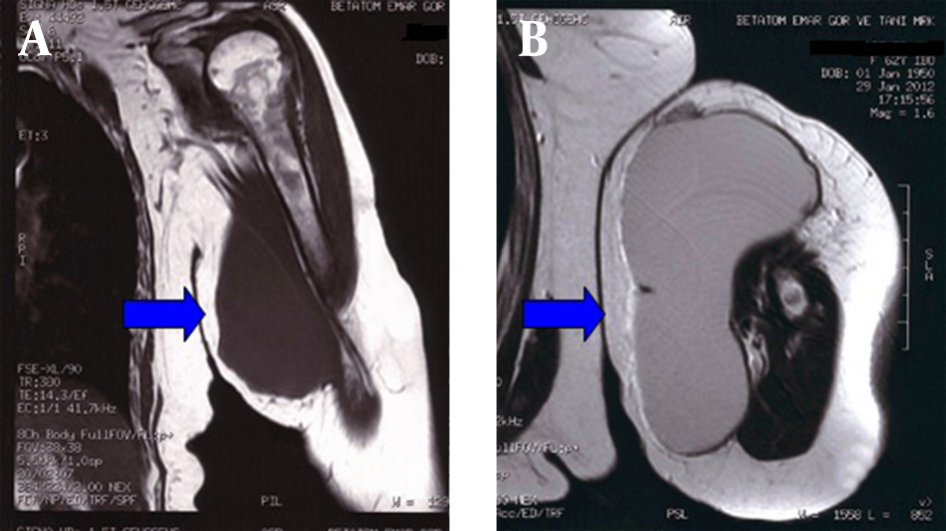
The Images of Left Arm Abscess in Coronal (a) and Axial Planes (b)

A 61-year old woman presented to our hospital with pain and a mass in her left arm. The patient's medical history revealed that the mass appeared approximately 6 months ago, and reached the size of a grapefruit in the past two months. She had a history of cervix cancer and was given radiotherapy and chemotherapy 3 years ago, and she was using methotrexate 10 mg/week, prednisolon 5 mg/day and hydroxychloroquine 200 mg/day for rheumatoid arthritis for the past 8 years. She had no history of trauma, fever or gastrointestinal infection. Her physical examination was normal except for the mass about 10x12 cm in the middle left arm. Upon admission, the patient did not have fever, and her axillary body temperature was 36.8 °C. Laboratory analyses revealed white blood cell count of 7.000/mm³ (82% polymorphonuclear leukocytes), hemoglobin level of 10.8 g/dL, erythrocyte sedimentation rate (ESR) of 37 mm/hour, and C-reactive protein (CRP) level of 4.2 mg/dL. Her ESR was 28 mm/h and CRP level was 1.3 mg/dL during control visit for rheumatoid arthritis approximately three months before. Since the patient had a positive malignancy history, magnetic resonance imaging of the left arm was performed and the mass was considered as an abscess that was then surgically drained (Figure 1).

Salmonella enterica spp. enterica was yielded in the culture of the abscess drainage material. Automated system VITEK2 (bioMerieux, Marcy I’Etoile, France) - GN-N91 card was used for the identification of the microorganism and antibiogram testing. Isolates were found to be susceptible to ampicillin, ceftriaxone, and trimethoprim-sulfamethoxazole; and resistant to cefuroxime and ciprofloxacin. Gruber Widal test was positive at 1/20 titer for Salmonella parathypi BO. According to antibiotic susceptibility test results, parenteral (intramuscular) ceftriaxone at a dose of 2 g/day was initiated. At the end of 2 weeks, it was switched to oral cefixime treatment that lasted an additional 2 weeks completing a total of 4 weeks of antibiotic therapy. At the end of the therapy, no clinical, laboratory and radiological pathology was detected.

## 3. Conclusions

Pyomyositis is the pyogenic infection of the skeletal muscles usually involving the lower, enteric fever, bacteremia, and chronic carriers (3, 4). In addition, Salmonellosis frequently causes infection limited to the gastrointestinal system after extremities, and rapidly progresses to develop an abscess. It usually presents with high fever and muscle pain (5). The most common cause of pyomyositis is Staphylococcus aureus (66-90 %), and Salmonella spp. is among the pathogens for reported only in cases series (6). Salmonellosis remains a major health problem especially in developing countries. Salmonella consists of approximately 1700 serotypes, and each serotype may cause gastroenteritis food poisonings, and generally is a self-limiting disease. Bacteremia occurs in 5-45% of the patients (7). It was reported that spread of microorganisms with bacteremia may lead to complications such as meningitis, brain abscess, septic arthritis, liver abscess, parotid abscess and muscle abscess (3, 4, 7). Focal or metastatic salmonellosis represents 6% of non-typhi Salmonella infections and occurs in risk groups and patients with structural abnormalities, such as valvular disease, aneurysms or atherosclerosis, biliary or urinary tract abnormalities, bone abnormalities or prostheses (1, 6).

Soft tissue infections are rarely seen and consists 6-12 % of focal Salmonella infections. Management of Salmonella abscess requires a combination of antibiotherapy, surgical drainage and eradication of the primary foci (6). Suppression of the immune system is a risk factor for extraintestinal Salmonellosis. Sickle cell anemia, diabetes mellitus, atherosclerosis, human immunodeficiency virus (HIV) infection, malignancy, chemotherapy, systemic lupus erythematosus and long term high dose steroid use are reported risk factors for invasive salmonellosis and bone-joint involvement (8-10). Salmonella is a well-known cause of endovascular infections such as mycotic aortic aneurysm in different anatomical regions. Atherosclerotic plaques containing a variety of phagocytic cells are assumed to be preferable for Salmonella, having ability to live and reproduce in macrophages (10). It is considered in our patient that this muscle abscess occurred as a result of infection in the atherosclerotic plaque during transient bacteremia.

In our patient, because of the underlying diseases, the antibiotic treatment was completed to 4 weeks with consecutive 3rd generation cephalosporins in order to prevent recurrence despite surgical drainage of the abscess. Reports of Salmonella isolates resistant to 3rd generation cephalosporins directed to new treatment options and quinolones were started to be preferred8. However, as seen in our patient, the occurrence of ciprofloxacin resistance, suggests alternative antibiotics such as carbapenems might come into prominence in the next years. Increasing antimicrobial resistance to fluoroquinolones and extended spectrum cephalosporins among clinical Salmonella isolates is becoming a serious problem, especially in Asia. The majority of antibiotics that demonstrate in vitro antimicrobial activity cannot cure Salmonella infections due to their limited intracellular penetration. Novel drugs with good both intracellular and extracellular antibacterial activity are urgently needed (10). Tigecycline has been shown to achieve high intracellular concentrations and exhibits a promising survival outcome compared with traditional ceftriaxone therapy in an animal model (11). In another animal study, ertapenem also was shown to be at least as effective as ceftriaxone (10). In the presence of conditions which suppress the immune system, low titers of positive serological tests may be seen and efforts should be made to the isolation of the agent. Salmonella infections should be considered in the differential diagnosis in endemic areas due to delayed or inappropriate treatments leading to serious complications.
